# Acute coronary syndrome following percutaneous closure of a coronary artery fistula: the essential anticoagulation management—a case report

**DOI:** 10.1093/ehjcr/ytag668

**Published:** 2026-09-09

**Authors:** Servoz Clément, Tamir El Mehdi, Bruguiere Eric, Combes Nicolas, Karsenty Clément

**Affiliations:** Cardiology Department of Rangueil Hospital, Toulouse University Hospital, 1 av. Pr. Poulhès, 31400 Toulouse, France; Cardiology Department of Rangueil Hospital, Toulouse University Hospital, 1 av. Pr. Poulhès, 31400 Toulouse, France; Congenital Cardiology Department, Clinique Pasteur, 45 av. Lombez, 31000 Toulouse, France; Congenital Cardiology Department, Clinique Pasteur, 45 av. Lombez, 31000 Toulouse, France; Department of Pediatric Cardiology, Centre Hospitalier Universitaire de Toulouse, 330 av. Grande-Bretagne, 31300 Toulouse, France; INSERM, UMR 1297, Institut des Maladies Métaboliques et Cardiovasculaires, 1 av. Pr. Poulhès, 31400 Toulouse, France

**Keywords:** Coronary artery fistula, Percutaneous closure, Antithrombotic therapy, STEMI, Thromboembolism, Case report

## Abstract

**Case summary:**

We report the case of a previously healthy 19-year-old woman with a large left coronary trunk–to–right atrium fistula, managed by percutaneous closure. Post-procedural antithrombotic therapy included apixaban and low-dose aspirin. One month later, the patient developed severe metrorrhagia with no identifiable organic cause, prompting unilateral modification of antithrombotic therapy without multidisciplinary discussion. Two months post-intervention, she presented with an acute ST-segment elevation myocardial infarction, likely resulting from thrombus formation at the prosthesis site. Coronary flow of the left anterior descending artery was restored by successful thromboaspiration without stenting.

**Discussion:**

This case highlights that any modification of antithrombotic therapy following percutaneous fistula closure must result from a thorough multidisciplinary discussion, carefully balancing bleeding risk against the potentially fatal consequences of thromboembolic events.

Learning points

- Effective management of antithrombotic therapy following percutaneous closure of coronary artery fistulas is critical to prevent thromboembolic complications.
- Tailored antithrombotic strategies should be considered in embolic STEMI based on the underlying cause and patient risk profile.

## Introduction

Coronary-cardiac fistulas are abnormal communications between coronary arteries and cardiac chambers or their afferent vessels, first described post-mortem by Kraus in 1865.^1^ Their incidence is estimated at 0.2%–0.6% of all congenital heart defects.^2^ Clinical manifestations are closely related to fistula size, which explains why the condition is often asymptomatic for years. Diagnosis relies on coronary angiography, CT scan, or magnetic resonance imaging. While small fistulas may close spontaneously, larger ones causing chamber enlargement or myocardial ischaemia require closure, either surgically or percutaneously. We report the case of a woman presenting with a large proximal coronary artery fistula between the LCT and the RA, treated by percutaneous closure and complicated 2 months later by an acute STEMI following discontinuation of antithrombotic treatment.

## Summary figure

**Figure:**
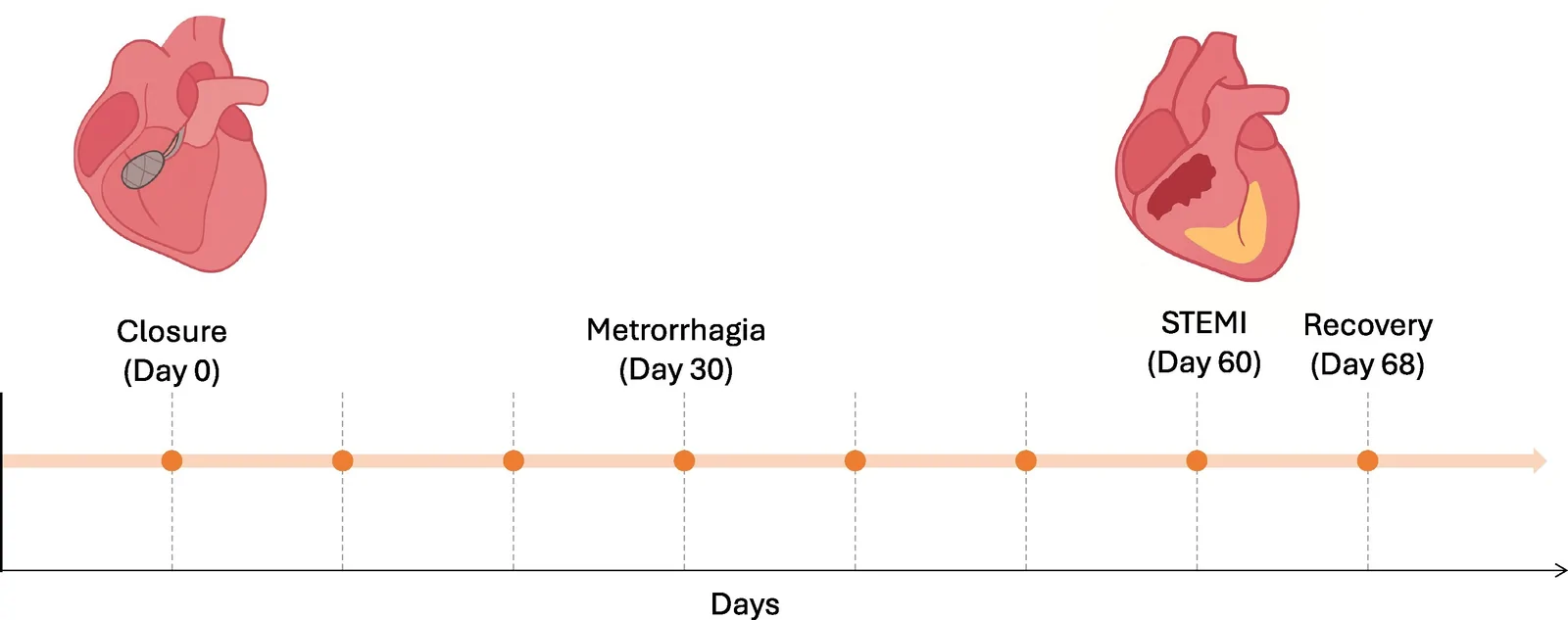
Percutaneous closure of the coronary artery fistula at Day 0, followed by severe metrorrhagia at Day 30, thromboembolic STEMI at Day 60, and subsequent recovery after successful coronary revascularization at Day 68.

## Case presentation

**Figure 1:**
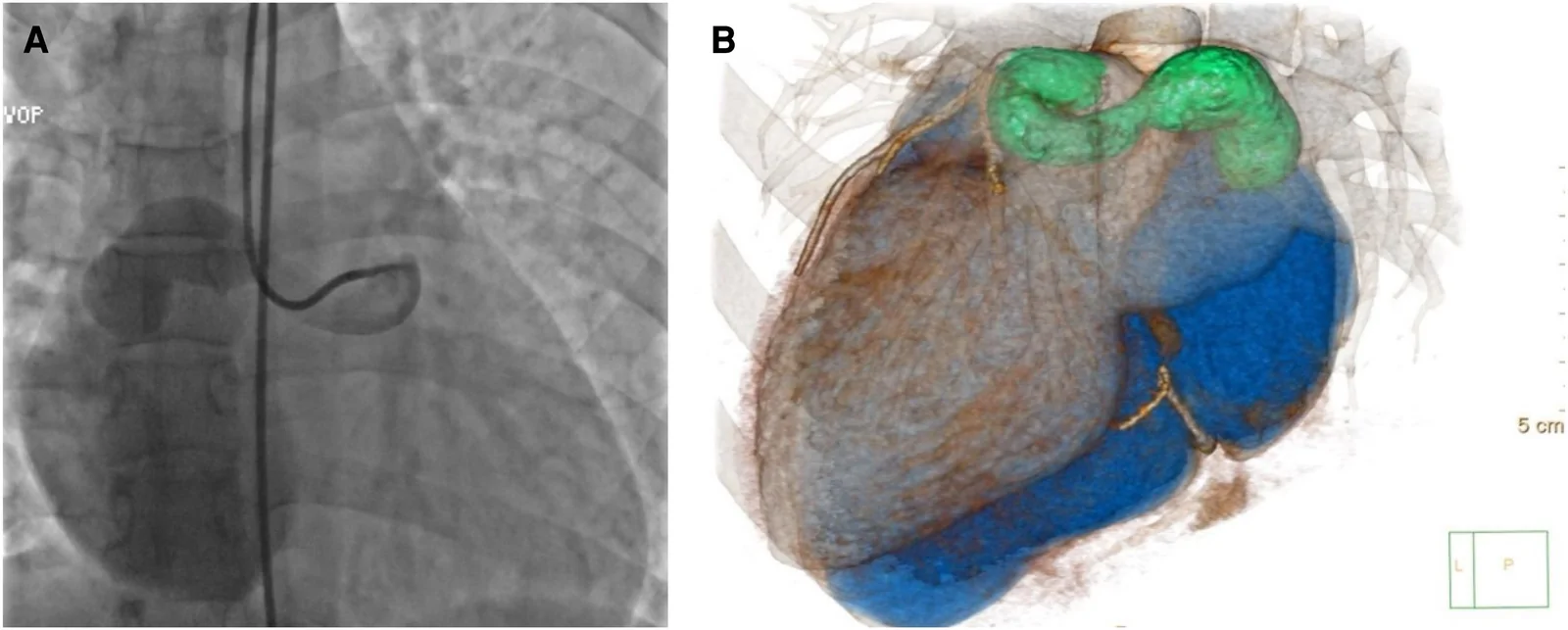
Large fistula between the left coronary trunk (LCT) and the right atrium (RA), measuring approximately 12–15 mm in diameter, associated with marked dilation of the LCT (17 mm) and enlargement of the RA. (*A*) Anterior view on coronary angiography showing the LCT/RA fistula. (*B*) Posterolateral 3D reconstruction from cardiac CT scan illustrating the fistulous tract (highlighted in green), with the right atrium and right ventricle (RA and RV) shown in blue.

**Figure 2:**
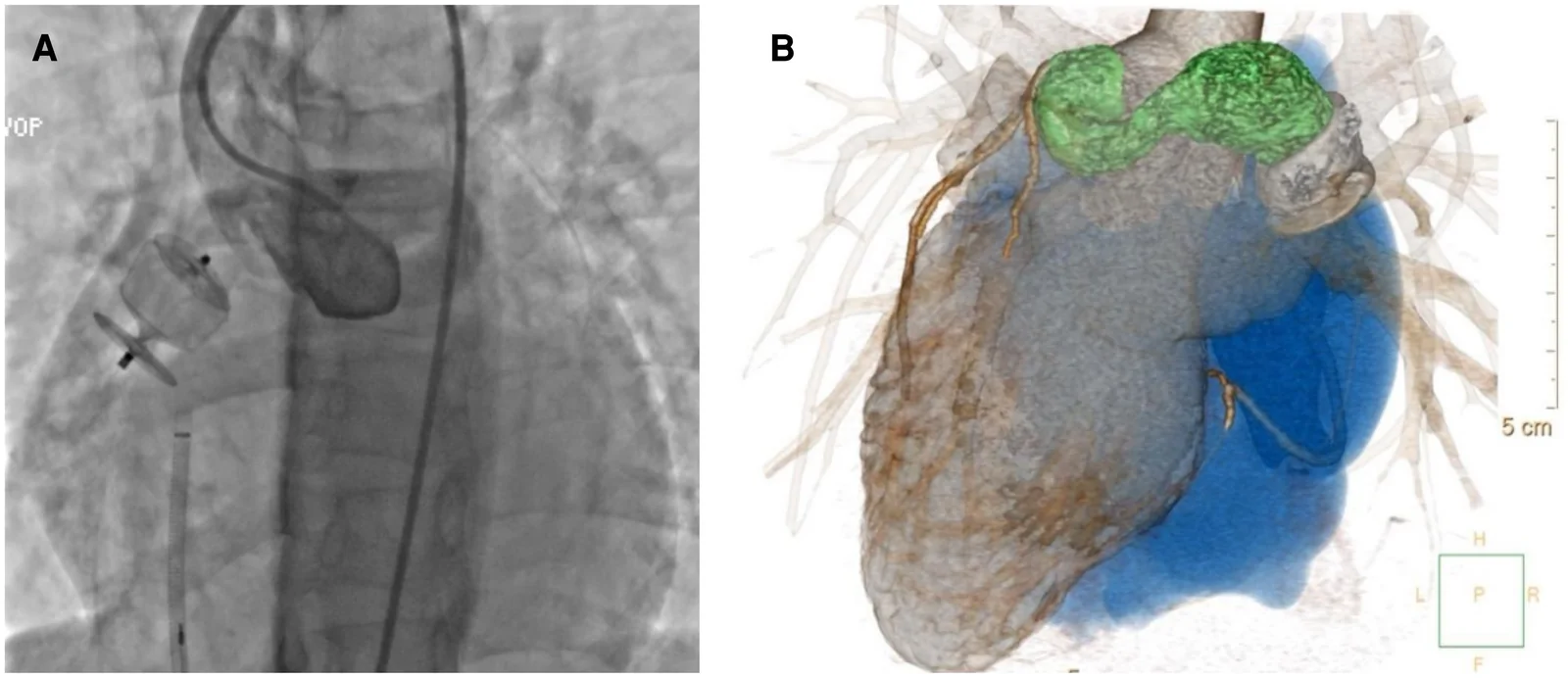
Percutaneous closure of the left coronary trunk (LCT) to right atrium (RA) fistula using a 20 mm amplatzer vascular plug II (AVP II), deployed on the RA side of the fistula. (*A*) Anterior view on coronary angiography demonstrating device placement. (*B*) Posterior view from 3D cardiac CT scan confirming the position of the vascular plug within the fistulous tract.

A 19-year-old previously healthy woman presented with chest pain, leading to the discovery of a large fistula between the left coronary trunk (LCT) and the right atrium (RA), measuring 12–15 mm in diameter. A continuous murmur was noted on clinical examination. Transthoracic echocardiography was inconclusive due to poor image quality. Imaging revealed significant dilation of the LCT (17 mm) and RA enlargement (*Figure 1*). Given the large fistula size, massive LCT dilatation (>10 mm), and chamber enlargement, a multidisciplinary team indicated occlusion. The fistula was successfully closed via a percutaneous transvenous approach using a 20 mm Amplatzer Vascular Plug II (AVP II, Abbott), deployed in the distal portion of the fistula on the atrial side (*Figure 2*). The procedure was uneventful, and the patient was discharged on apixaban 5 mg twice daily and aspirin 100 mg daily.

One month later, she presented to gynaecological emergency with profuse metrorrhagia. Clinical and paraclinical investigations were normal, with no identifiable gynaecological cause. Aspirin was discontinued and apixaban was reduced to 2.5 mg twice daily. This dose reduction was guided by the patient's low body weight (45 kg), meeting one standard dose-reduction criterion for apixaban (weight ≤ 60 kg, age ≥ 80 years, or creatinine ≥ 1.5 mg/dL). Although only one criterion was formally met, this adjustment was made by the gynaecologist without any multidisciplinary discussion—a critical gap in care and a key learning point of this case. Tranexamic acid and parenteral iron supplementation were initiated in parallel.

**Figure 3:**
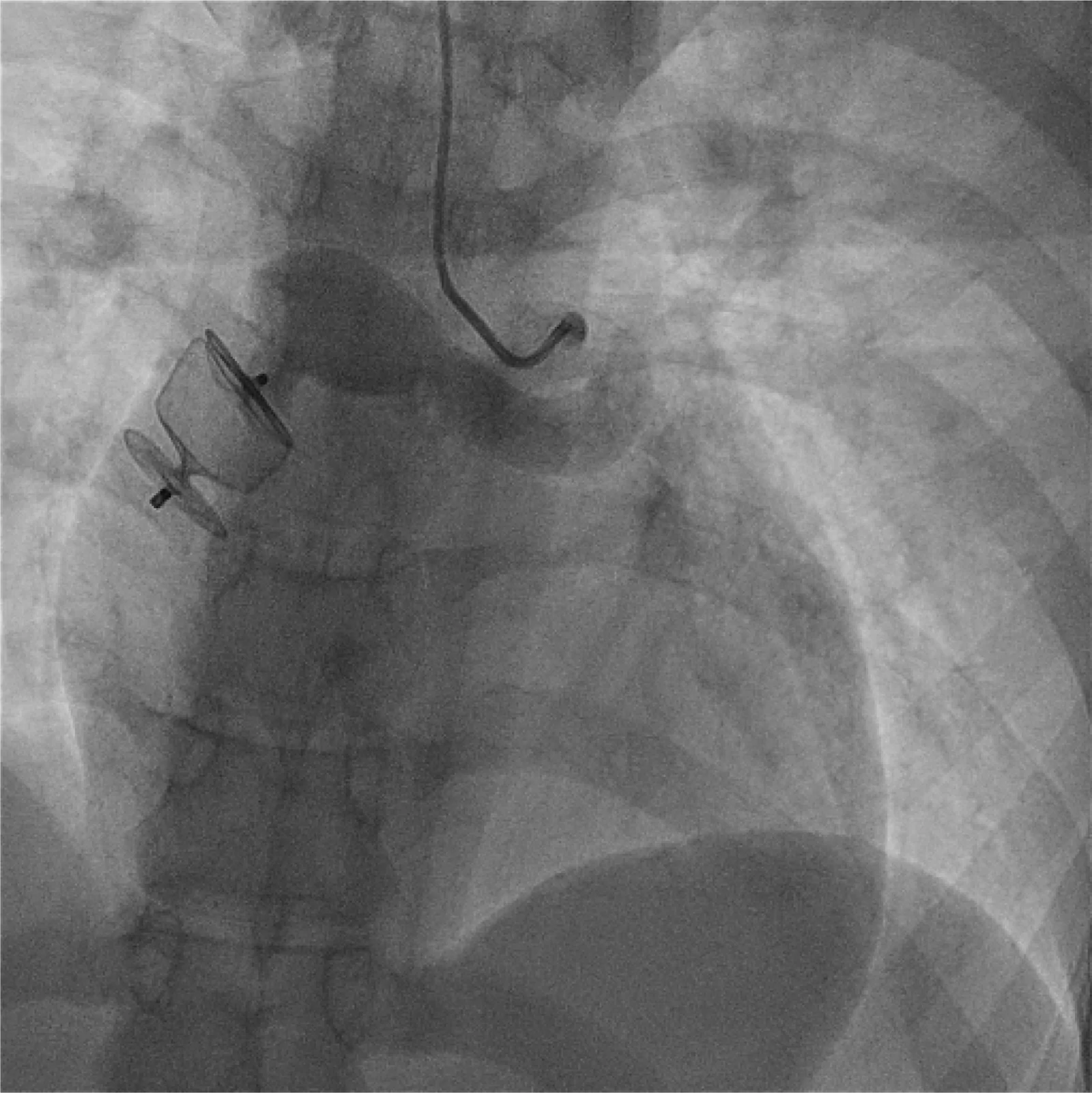
Coronary angiography showing the presence of thrombus adherent to the vascular prosthesis.

**Figure 4:**
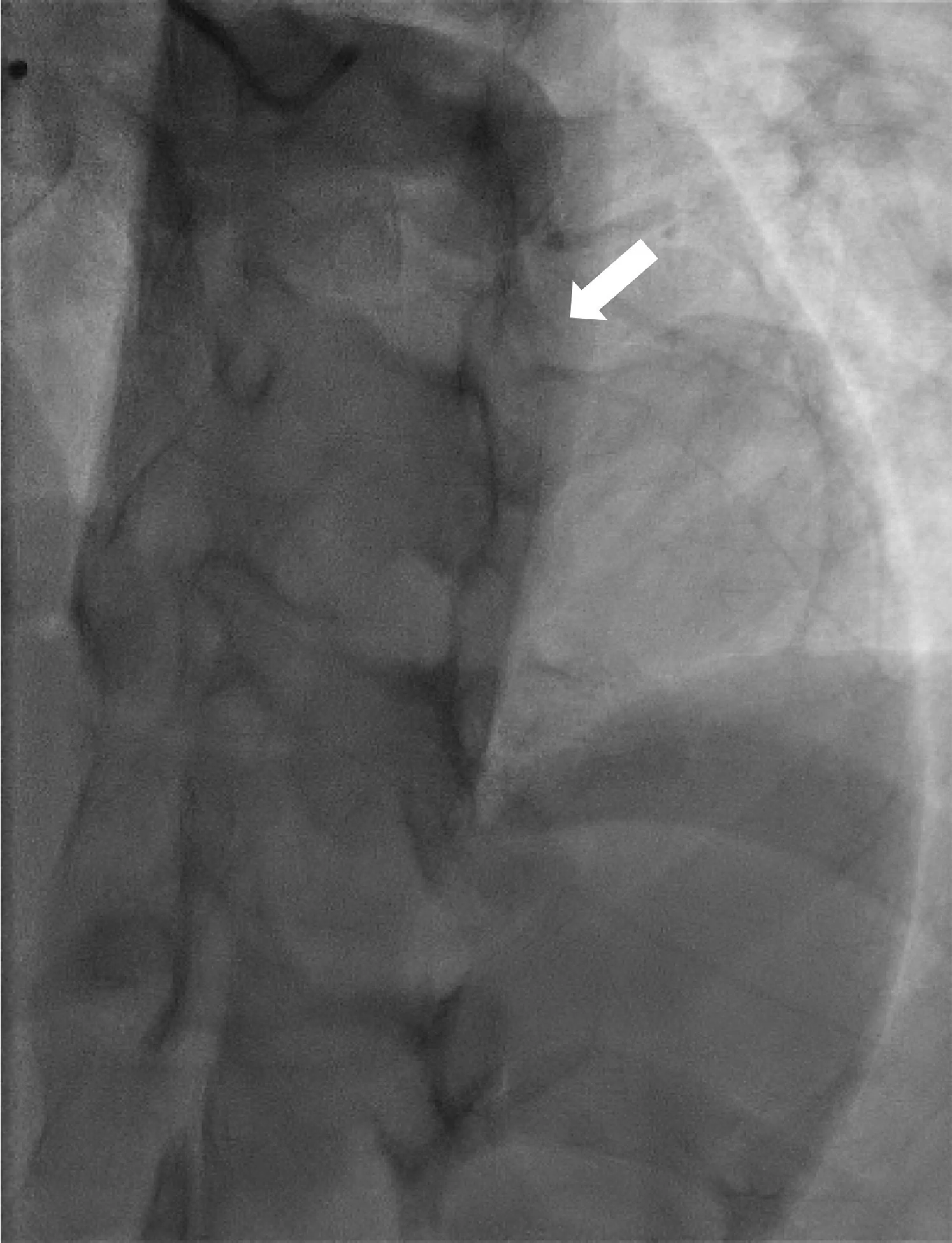
Occlusion of the left anterior descending artery caused by thromboembolic obstruction, as visualized on coronary angiography.

**Figure 5:**
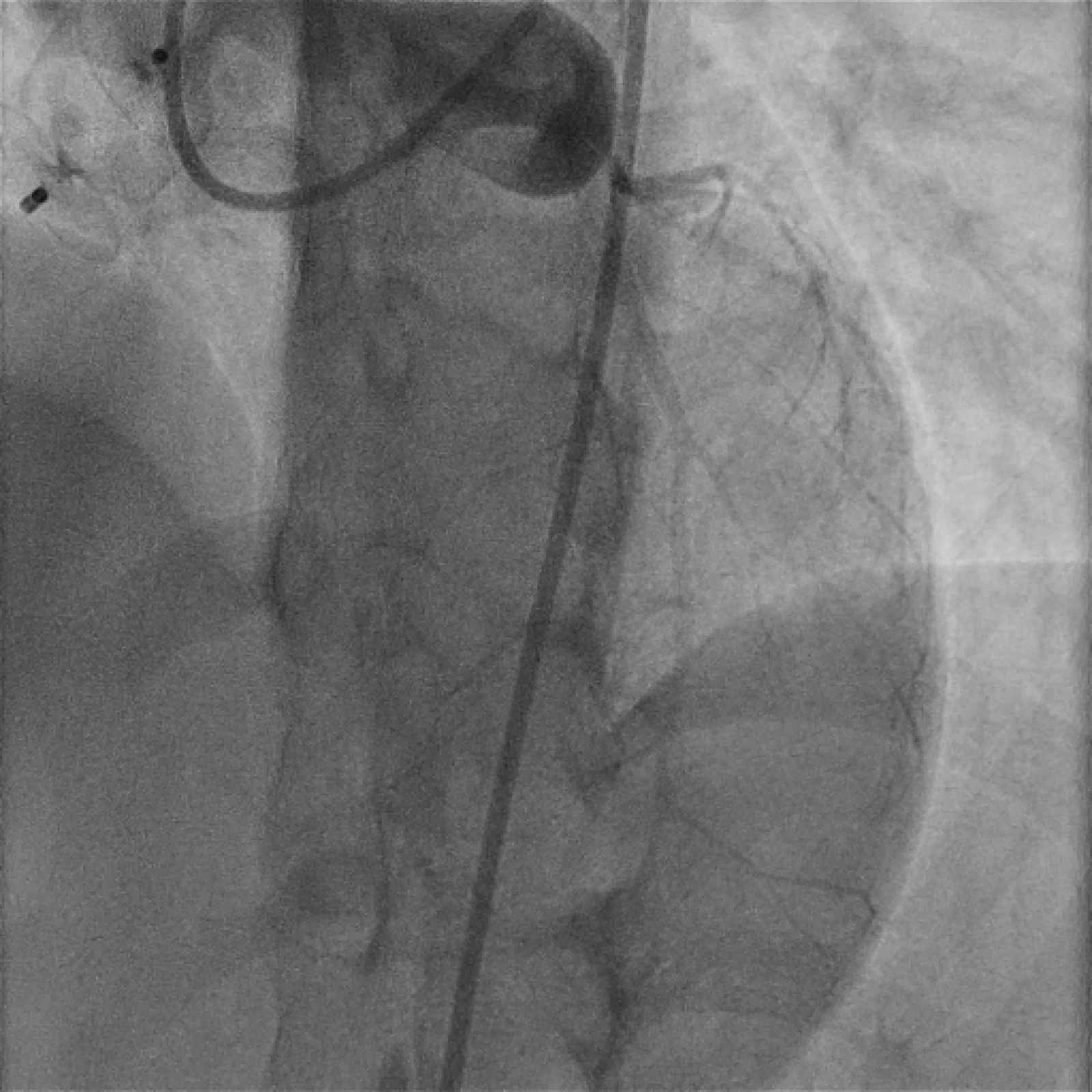
Repermeabilization of the left anterior descending artery following successful thromboaspiration, with restoration of coronary blood flow.

One month later, the patient presented to the emergency department with typical constrictive chest pain radiating to the left arm. ECG revealed ST-segment elevation in anterior leads, confirming STEMI. Coronary angiography demonstrated LAD occlusion from embolic occlusion (*Figures 3–4*, Supplementary material online, *Video S1*). Reperfusion was successfully achieved by thromboaspiration without stenting (*Figure 5*, Supplementary material online, *Video S2*). Given the absence of underlying coronary artery disease, thromboembolism was considered the most likely aetiology.

Initial echocardiography showed preserved LVEF with septo-apical kinetic abnormality and no thrombus (see Supplementary material online, *Video S3*). The vascular plug was visualized within the right atrium. Cardiac CT confirmed thrombus at the prosthetic device. At 24 h, LVEF declined to 30% with akinesia of the entire apical region extending to the anteroseptal wall, recovering to 43% at 8 days.

Given the confirmed thromboembolic event and prior interruption of antithrombotic therapy, anticoagulation was switched to warfarin (INR target 2–3) combined with aspirin, planned for 6 months. The decision to use a vitamin K antagonist rather than a DOAC was motivated by several considerations: the need for monitorable anticoagulation intensity in confirmed device-related thrombosis, the ability to titrate INR to a precise therapeutic target with real-time monitoring, an established reversal strategy in case of recurrent haemorrhage, and the absence of validated evidence for DOACs in this specific indication. While DOACs may offer equivalent efficacy with a more favourable bleeding profile in other thromboembolic contexts, the lack of data following CAF closure led us to favour a monitorable regimen, consistent with the pragmatic framework proposed by Gowda *et al*. [4]. Following gynaecological consultation, desogestrel-based contraception was initiated to mitigate the risk of recurrent heavy menstrual bleeding.

## Discussion

Coronary-cameral fistulas account for approximately 90% of coronary artery fistulas, occurring when a coronary artery terminates directly into a cardiac chamber. According to a meta-analysis by AM Said, 69% originate from the left coronary artery, are unilateral in 80% of cases, and drain into the venous circulation in 90% of cases.^2,3^ Pathophysiologically, they cause ‘coronary steal,’ driven by the diastolic pressure gradient between the high-pressure coronary artery and the low-pressure receiving chamber. The extent of the shunt depends on the diameter and length of the fistulous tract.

Small fistulas generally do not require treatment given frequent spontaneous closure. In larger fistulas, percutaneous closure has become the preferred approach since the 1980s, with success rates exceeding 90%, reported mortality below 1%, and minimal morbidity.^2^ Long-term follow-up is essential to monitor for myocardial ischaemia, persistent shunting, and evolution of coronary artery dilation. Potential complications include myocardial infarction, device embolization, and fistula recanalization, making antithrombotic therapy advisable following percutaneous closure.^4,5^

There are currently no established guidelines regarding the optimal antithrombotic regimen following percutaneous CAF closure. Gowda *et al*.^4^ provide a valuable framework for this decision: oral anticoagulation, with or without concomitant antiplatelet therapy, is justified in cases with large fistulas or significant residual thromboembolic risk, particularly during the device endothelialization phase, typically spanning the first three to six months post-implantation. This prothrombotic window, driven by incomplete device endothelialization and disruption of laminar flow, justifies anticoagulation as the cornerstone of post-procedural management. The addition of short-term antiplatelet therapy may further reduce platelet-mediated thrombus formation on the device surface, provided that bleeding risk is carefully and regularly reassessed in a multidisciplinary setting. In our patient, the initial regimen of apixaban combined with low-dose aspirin was consistent with this evidence-based approach, and the thromboembolic event following its discontinuation further underscores its critical importance.

This case illustrates that any modification of antithrombotic therapy after percutaneous device implantation must result from multidisciplinary discussion integrating cardiology, gynaecology, and haematology expertise, in order to individually balance thromboembolic and bleeding risks.

## Conclusion

Coronary artery fistulas are rare anomalies, often discovered incidentally, that may remain asymptomatic before leading to myocardial ischaemia, significant left-to-right shunting, or chamber dilation and heart failure. Cardiac CT is essential for accurately identifying the origin, course, and drainage site of the fistula and for guiding therapy. Endovascular treatment is highly effective but requires strict antithrombotic management to prevent device-related thrombosis, which may result in myocardial infarction if inadequately controlled. This case further illustrates that any modification of antithrombotic therapy following percutaneous device implantation must be made exclusively within a multidisciplinary framework, integrating cardiology, gynaecology, and haematology expertise, in order to avoid potentially fatal thromboembolic complications.

## Lead author biography


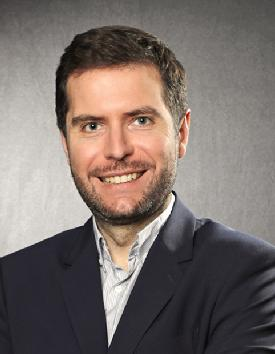
Dr. Clément Servoz is an interventional cardiologist based in Toulouse, France, working at Toulouse University Hospital. His clinical expertise focuses on coronary artery disease, coronary physiology assessment, and percutaneous coronary interventions.

## Supplementary Material

ytag668_Supplementary_Data

## Data Availability

All data underlying this article are available as part of the article. No additional source data are required.
