# Supplementary material for: Acute coronary syndrome following percutaneous closure of a coronary artery fistula: the essential anticoagulation management—a case report
Source: Eur Heart J Case Rep. 2026 Sep 9;10(9):ytag668. doi: 10.1093/ehjcr/ytag668 (PMC13614258; doi:10.1093/ehjcr/ytag668)
Supplement: Supplementary file 1 [file ytag668_supplementary_data.zip › Supplementary_legends.docx]

Video 1 : Coronary angiography showing occlusion of the left anterior descending artery due to thromboembolic obstruction.

-Video 2 : Final angiogram showing revascularization with TIMI 3 flow of the left anterior descending artery after successful thromboaspiration.

-Video 3 : Transthoracic echocardiography showed preserved left ventricular ejection fraction and a septal and apical wall motion abnormality.

-Supp. figure : Electrocadiogram showing anterior ST-segment.
